# My Sadness – Our Happiness: Writing About Positive, Negative, and Neutral Autobiographical Life Events Reveals Linguistic Markers of Self-Positivity and Individual Well-Being

**DOI:** 10.3389/fpsyg.2018.02522

**Published:** 2019-01-08

**Authors:** Cornelia Herbert, Eileen Bendig, Roberto Rojas

**Affiliations:** ^1^Institute of Psychology and Education, Applied Emotion and Motivation Psychology, Ulm University, Ulm, Germany; ^2^Institute of Psychology and Education, Clinical Psychology and Psychotherapy, Ulm University, Ulm, Germany; ^3^Institute of Psychology and Education, University Psychotherapeutic Outpatient Clinic, Ulm University, Ulm, Germany

**Keywords:** expressive writing, emotion expression, self, positivity bias, negativity bias, social cognition, self-reference, we-reference

## Abstract

**Objective:** Narratives of autobiographical events contain rich information about an individual’s private experience, his/her deepest thoughts, feelings, and emotions. The present study investigates linguistic markers of emotion expression and subjective well-being in adults during one session of positive, negative, and neutral expressive writing. Participants (*N* = 28 healthy participants, *N* = 7 adults with depressive symptoms), all native speakers of German were instructed to write expressively about personally relevant autobiographical life events of negative, positive, and neutral content.

**Methods:** Quantitative text analysis was performed to determine the amount of emotional words, first person pronouns (singular vs. plural), and cognitive function words used in positive, negative, and neutral narratives and to examine the potency of these classes of words as linguistic markers of positivity/negativity, self-reference, and cognitive reappraisal. Additionally, the relationship between expressive writing and subjective well-being was explored by assessing changes in self-reported psychosomatic symptoms and in bodily and emotional awareness immediately after positive, negative, and neutral writing.

**Results:** Regarding healthy participants, negative narratives contained significantly more negative emotional words than positive or neutral narratives. However, negative narratives also contained more positive emotional words compared to negative emotional words in positive narratives. Moreover, negative narratives contained more cognitive function words than positive narratives, suggesting that healthy participants tried to reappraise negative experiences while writing about negative personal life events. Positive narratives were characterized by an increased use of positive words and of pronouns of the first person plural (“we”), supporting a positivity bias and an extension of self-reference from first person singular to plural (we-reference) during positive expressive writing. Similarly, writing about neutral events was characterized by a positivity bias. Although based on descriptive analysis only, preferential use of positive words and cognitive function words in negative narratives was absent in participants reporting depressive symptoms. Positive, negative, and neutral expressive writing was accompanied by differential changes in bodily sensations, emotional awareness, and self-reported psychosomatic symptoms in all participants.

**Discussion:** The findings are discussed with respect to previous research, a self-positivity bias, and a universal positivity bias in language use highlighting the relevance of these biases as markers of subjective well-being.

## Introduction

Language is a powerful tool of human communication; it constitutes an important medium for conveying thoughts, feelings, emotions, and actions and for reflecting about them (Chomsky and Smith, 2000). In particular during writing, we express emotions by putting feelings into words. In turn, the words we use may shape our feelings, mood, and affective and cognitive state. Although the exact mechanisms by which language and emotions interact and influence each other are still a matter of ongoing scientific research [e.g., see this research topic and Herbert et al. (2018); for an overview], previous research has left no doubt that labeling one’s own feelings by means of words as well as writing expressively about them can have positive effects on individual well-being (e.g., Pennebaker and Chung, 2007; Torre and Lieberman, 2018; for overviews). For instance, considering experimental research on emotional word processing, there is evidence that written words can induce emotions in the sender and the perceiver of a message and shape emotion perception of non-verbal stimuli instantly by modulating brain activity in the visual cortex and in brain structures such as the amygdala involved in fear and emotion processing (Lieberman et al., 2007; Herbert et al., 2008, 2013, 2018; Scott et al., 2009; Herbert et al., 2009). The results suggest that the words we see, read, hear, and use for emotion expression can modulate how we perceive and regulate emotions during emotional processing. This can occur without us being fully aware of the elicited perceptual changes and bodily responses.

### Expressive Writing as a Means of Emotion Expression and Individual Well-Being

In line with this suggestion, several studies show that asking participants to write expressively about traumatic or emotionally distressing autobiographical events can have positive effects on physical health and psychological well-being (e.g., Pennebaker and Chung, 2007; Lepore and Kliewer, 2013 for an overview). Regarding psychological well-being, prominent effects have been reported with regards to changes in self-reported depressive symptoms (e.g., Krpan et al., 2013) and self-reported anxiety (Al-Mosaiwi and Johnstone, 2018). Effects have been documented in studies investigating college students, community samples, and in individuals at risk or suffering from psychosomatic diseases, trauma (physical and psychological), or psychiatric disorders [Frisina et al. (2004) for an overview]. Concerning anxiety, particularly strong effects have been reported in individuals scoring high on scales of emotional expressivity (e.g., Niles et al., 2014).

Regarding the content and quantity of expressive writing, converging evidence exists that especially writing about highly emotionally distressing events can have psychological and physiological health benefits (Baikie and Wilhelm, 2005; Frattaroli, 2006). Hence, the typical expressive writing protocol in most studies asks participants to write down their deepest thoughts and feelings about their most traumatic experience (Pennebaker and Beall, 1986) over the course of 3–4 days. More recent research shows that positive health effects of expressive writing are not restricted to writing about negative or highly traumatic events: writing about positive autobiographical events can have similar favorable and positive effects on individual well-being; however, only if the writer refrains from re-analyzing the positive event in analytical terms (Burton and King, 2004; Lyubomirsky et al., 2006; Lewandowski, 2009).

Regarding immediate effects of expressive writing on individual well-being the literature is mixed and results seem to vary more strongly across measures and domains of well-being (Sloan and Marx, 2018). Nevertheless, some studies found that already one writing session can be sufficient to provoke changes in self-reported mood and psychosomatic symptoms (Baikie and Wilhelm, 2005; Frattaroli, 2006). However, immediate effects can be in opposition to the long-term effects. As far as writing about negative events is concerned, the immediate impact of expressive writing is usually a short-term increase in distress, in negative mood, and in physical symptoms and a short-term decrease in positive mood (cited from Baikie and Wilhelm, 2005, p. 339; Frattaroli, 2006). Similar changes may appear after writing analytically about positive events. Nevertheless, when repeated within a few hours across the day, expressive writing about demanding life events may have the same long-term health benefits as writing consecutively for 15–20 min across 3–4 days (Pennebaker and Chung, 2007).

Theoretically, several explanations have been put forward to account for these findings. Both, psychological as well as physiological suggestions have been made to explain the effects of writing on individual well-being. Among the psychological explanations, repeated exposure with the emotional distressing event, cognitive reappraisal, self-disclosure, as well as cognitive restructuring (forming a coherent story) have been proposed as mechanisms underlying the relationship between expressive writing and self-reported emotional health (Pennebaker and Chung, 2007; for an overview). Whereas repeated exposure may hold true as a mechanism of repeated expressive writing, cognitive reappraisal, self-disclosure, and cognitive restructuring may constitute potential mechanisms already effective after one expressive writing session. These mechanisms have been linked mainly to expressive writing about negative events. Although writing expressively about negative and positive events may have emotional and cognitive aspects in common, the mechanisms underlying expressive writing about positive events may differ from those underlying negative expressive writing (Wing et al., 2006). Regarding the mechanisms specific to expressive writing about positive autobiographical events only few speculations have been made so far. For instance, it has been hypothesized that writing about positive events may lead to an instantaneous increase in positive mood and in the long-run promote emotion processing skills typically associated with improved social cognition and personality traits such as emotional intelligence (e.g., Wing et al., 2006).

### Linguistic Markers of Emotion Expression and Individual Well-Being

Although the effects of writing on well-being are undisputed in the literature, it is still a matter of ongoing research how exactly these health-related cognitive processes and proposed mechanisms of expressive writing (e.g., cognitive reframing, reappraisal, self-disclosure) do manifest in writing: in particular there is ongoing research on how psychological variables can be inferred through linguistic markers and hence by the way we write and use different types of words during writing. In search of specific linguistic markers, fully automated word count algorithms based on standardized word dictionaries have been developed. These allow reliable quantification of words according to pre-classified semantic and grammatical word categories. Studies applying these algorithms to expressive writing found considerable inter- and intra-individual differences in word use within and across writings differing in emotionality (positive, negative neutral) (e.g., Frattaroli, 2006; Pennebaker and Chung, 2007; for an overview).

#### Negativity Bias and Self-Reference

Most evidence that the use of certain words can provide insight into the writer’s feelings, his/her self and private thoughts, and experiences – thereby allowing predictions about well-being – comes from expressive writing studies investigating individuals suffering from somatic and mental disorders (e.g., Frisina et al., 2004). Regarding mental disorders, linguistic analysis has been provided by studies investigating thought disorders such as schizophrenia (e.g., Buck and Penn, 2015; Hong et al., 2015) and affective disorders, most notably depression (e.g., Krpan et al., 2013; Al-Mosaiwi and Johnstone, 2018; Tackman et al., 2018). Regarding depression a valid finding across studies is a rigorous use of first-person singular pronouns (“I”, “me”, “my”) and of negative emotion words during negative expressive writing about personal live events (Pennebaker and Chung, 2007; Tackman et al., 2018). The results fit with cognitive models of depression. These suggest (a) an increase in self-focused attention and (b) a negativity bias in the evaluation of emotional information as key symptoms of the depressive disorder (e.g., Pyszczynski and Greenberg, 1987). Thus, use of first person pronouns singular and use of negative emotional words may indeed indicate depression-related changes in emotional appraisal and in self-focus. What is currently unknown is whether an increased use of first person pronouns singular as well as of negative words is also apparent in healthy subjects during expressive writing of negative compared to neutral or positive life events; an observation that would support the view that word use is context-dependent. In support of this latter view, studies investigating natural language use suggest that an increased use of self-related and negative words seems to be depression-specific during expressive writing but not during daily conversation or everyday spoken language (e.g., Rodriguez et al., 2010). Therefore, linguistic markers may be both, context-dependent state markers and context-independent trait markers.

#### Positivity Bias and Self-Reference

Rigorous use of negative and self-related words during negative expressive writing contrasts with a universal positivity bias in human language, the latter reflecting an overall tendency toward over-representation of positive content in most if not all languages (e.g., Dodds et al., 2015). Indeed, although still not fully understood, positive words seem more prevalent in many languages, more readily learned and also more diversely used than negative words even if differences in word-frequency are controlled for. A processing bias toward positive words – well matched with negative or neutral words in linguistic dimensions (e.g., emotional arousal, word frequency, and word-length) – has also been reported in experimental studies on word reading: the results indicate that in healthy individuals positive content is preferentially processed under a number of task conditions including silent reading, lexical decision, and active attention focus (e.g., Herbert et al., 2008; Kissler et al., 2009; Scott et al., 2009). Moreover, when combined with pronouns, word processing studies in healthy individuals even support a self-positivity bias: for instance, during reading, nouns paired with “my” are encoded more deeply, evaluated more positively, and remembered better than nouns with a reference to another person (“his”) (e.g., Herbert et al., 2011; Fields and Kuperberg, 2016; Weis and Herbert, 2017). The empirical observations agree with the idea of a universal positivity bias and with findings of mood congruent processing, positive mood being the normal experience in healthy subjects (Diener et al., 2018). Whether healthy people also show a tendency to positivity or even toward self-positivity when expressing feelings during writing and whether this bias is universal or context-dependent has not been examined yet.

Finally, pronouns and emotional words are not the only linguistic markers providing information about a person’s internal mental self-representations. Cognitive function words constitute a special grammatical class of words providing information about how a person organizes, elaborates structures, appraises, and interprets the emotional content he/she is writing about. Previous studies found that the use of cognitive function words can significantly increase during expressive writing, particularly when participants attempt to transform and explain their negative life events. This kind of cognitive reappraisal of specifically negative experiences can be reflected by both, the use of cognitive function words, cognitive mechanism, causation, and insight words in particular (Tausczik and Pennebaker, 2011; Alparone et al., 2015) and by a higher ratio of positive vs. negative emotional words in negative narratives than negative vs. positive emotional words in positive narratives (McAdams et al., 2001). In linguistic terms, unlike nouns or adjectives cognitive function words mainly express relationships within a sentence for why as a class of words they are especially interesting for making inferences about how people interpret and interrelate the content they are writing about.

### Aim of the Present Study

The aim of the present study was to integrate various findings from previous expressive writing studies in order to clarify open questions regarding the relationships between linguistic markers, effects of short-term expressive writing and individual well-being. Following this endeavor, an experimental set-up was chosen in which healthy participants (*N* = 28) were asked to write expressively about positive, negative, and neutral autobiographical experiences under well-controlled laboratory conditions. Participants were invited only once which allowed us to investigate whether emotion expression, word use, and subjective well-being changes in each subject immediately as a function of the emotionality of the expressive writing context after just one single writing session. In particular, we assessed differential use of emotional words (positive words vs. negative words), pronouns (referential pronouns, first person singular vs. plural), and cognitive function words across positive, negative, and neutral narratives in order to elucidate the roles of these words as linguistic markers of positivity/negativity, self-reference, and cognitive reappraisal. Furthermore, although exploratory, a small sample of *depressive participants* (*N* = 7) was included in the study to explore the specificity of the positivity vs. negativity bias in expressive writing. Moreover, self-reported changes in mood, psychosomatic symptoms, bodily sensations, and subjective feelings were assessed in all participants (healthy participants and depressive participants) to determine immediate effects of positive, negative, and neutral writing on major components of subjective well-being. Finally, all participants (healthy participants and depressive participants) were asked about their willingness to self-disclose and their concerns about providing insight into personal experiences and feelings during writing.

In summary and as outlined in detail above, there are at least four questions from previous writing research that the present study aims to answer: First (1), extend previous results to languages other than English. Second (2), extend previous results of expressive writing on subjective well-being to the analysis of short-term effects. Third (3) and fourth (4), examine whether use of certain word categories in positive and negative narratives may be stable indicators of emotion expression including biases such as the self-positivity and explore the extent to which the use of certain word categories is different during depression. In line with these research questions and previous research, the following hypotheses were tested. The first hypothesis tested was that use of negative and positive emotional words will differ as a function of the emotional content, i.e., we expected use of negative words to be significantly more pronounced during negative expressive writing than during positive expressive writing or during writing about neutral events. Next, differences in the overall use of positive vs. negative words during negative and positive and neutral expressive writing were investigated to determine the hypothesis of a positivity bias in healthy participants and its potential absence in depressive participants. In line with this, the use of first person singular vs. first person plural pronouns and the use of cognitive function words were investigated to test the hypotheses that (a) the degree of self-focus and (b) the extent to which writers interrelate and reflect about the writing content differ when expressing feelings during positive, negative, and neutral writing. As mentioned above, whether healthy people do show a tendency to positivity or even toward self-positivity when expressing feelings during writing and whether this bias is absent in depression has not been explored in detail in previous studies in the context of expressive writing and within subjects and across positive and negative expressive writing. Finally, the hypothesis was tested that putting feelings into words during just one single session of positive and negative expressive writing may immediately provoke perceivable changes in bodily sensations and subjective feelings.

## Materials and Methods

### Participants

In total, *N* = 28 healthy adults (mean age: 24 years; 20 women and 8 men), all native speakers of German participated in the study. In addition, seven adults (mean age: 34.14 years; 6 women and 1 man) with former diagnosis of major depression (*N* = 6) and currently suffering from mild depression (*N* = 7) were included. Exclusion criteria for the sample of healthy participants were reports of prior or acute depression or reports of any other acute or chronic psychiatric or neurological disorder. Presence and severity of acute depressive symptoms were assessed via standardized self-report questionnaires including the Beck Depression Inventory (BDI-II; Hautzinger et al., 2006). Difficulties in emotion expression as well as participants’ concerns about self-disclosure during writing were also assessed to control for individual differences in verbal emotion expression and in the willingness to report one’s own feelings and experiences. In addition, self-reported anxiety was assessed with the Spielberger State Trait Anxiety Inventory (Spielberger, 1983) to control for interindividual differences in anxiety and for comorbidity between depressive symptoms and anxiety. To evaluate changes in positive and negative affect after each of the writing conditions (positive, negative, neutral), participants filled in the Positive Affect and Negative Affect Schedule (PANAS, Watson et al., 1988) prior to and after each writing condition. Sociodemographic variables including mean scores of depression (BDI-II), trait and state anxiety (STAI), and mean scores of positive (PA) and negative affect (NA) assessed prior to the writing conditions are reported in Table 1 for the group of healthy participants and the depressive participants.

**Table 1:** Self-report data, age, and gender of the participants (group means and standard errors).

	Healthy participants	Depressive participants
Sample size	28	7
Mean age	23.67 (1.16)	34.14 (2.70)
Gender	20 Women	6 Women
	8 Men	1 Man
BDI-II (Depression)	2.85 (0.57)	13.66 (1.87)
STAI (Anxiety)		
State	41.64 (2.64)	41.33 (0.29)
Trait	44.68 (3.06)	48.50 (0.34)
PANAS		
PA (positive affect)	30.29 (1.12)	22.86 (2.08)
NA (negative affect)	12.00 (0.67)	15.71 (1.40)


*Values are illustrated separately for the group of healthy participants and the group of depressive participants.*

### Expressive Writing Paradigm

In the present study a within-subject design was chosen. In total, *N* = 84 narratives (1 positive, 1 negative, and 1 neutral) were obtained from *N* = 28 healthy participants and *N* = 21 narratives (1 positive, 1 negative, and 1 neutral) were obtained from *N* = 7 depressive participants. Narratives of each participant were assessed in one session under standardized and well-controlled laboratory conditions. Expressive writing instructions for the positive, negative, and neutral narratives were adapted from Pennebaker and colleagues expressive writing recommendations and translated into German. In line with previous expressive writing studies, participants were instructed to really let their feelings go and to write honestly about their deepest emotions, feelings, and thoughts for at least 15 min and no longer than 20 min without worrying about spelling or grammar (e.g., Pennebaker, 2010). The order of emotional writing (positive, negative, neutral) was counterbalanced across participants. The neutral writing condition was always being offered as the second writing condition. In line with Chung and Pennebaker (2008) there were 10-min breaks between each writing condition. Akin to previous studies, participants were free to choose which of the most positive, negative, or neutral autobiographical life events they would like to write about.

After each writing, participants completed a post-writing-questionnaire and filled in the state version of the PANAS to assess writing-related changes in PA and NA. The post-writing questionnaire was adapted from the Physical Symptom Scale (PSS; Pennebaker, 1982) and captured participants’ experience of psychosomatic symptoms (e.g., tachycardia, stomach-ache, headache, fainting, and breathlessness), changes in psychophysiological bodily sensations (e.g., coldness, sweating hands, heart beats, and nervousness) and included additional questions on changes in emotional awareness for feelings (e.g., “I am feeling … sad/guilty/happy/confident/anxious”). Answers had to be given on 5-point-Likert-scales ranging from 1 (not at all) to 5 (very much). As a manipulation check, participants were asked to indicate for each writing, again on 5-point-Likert-scales, the degree to which they wrote in emotional and personal terms (e.g., “I wrote about a topic that matters to me”), the degree to which they were concerned about privacy and self-disclosure (“Did feeling safe and private influence your writing?”), and how difficult it was to express one’s feelings during writing^1^.

### Procedure

Participants were seated in a quiet, comfortable testing room where all testing and recoding took place. Participants were questioned about their health and gave informed consent. Consent obtained from all participants was both written and informed. Afterward participants received a booklet for writing down their narratives in a paper–pencil format. The booklet consisted of six pages (cover, demographic data, writing instruction, blank pages, and post-writing-questionnaire). The study was approved by the local ethics committee of the University of Ulm^2^. At the end of the experimental session, participants were fully debriefed about the purpose of the study and were reimbursed with 8€ for study participation. In addition, if desired they could talk to a professional psychotherapist in case that expressive writing about negative life events was too challenging or emotional.

### Analysis and Coding of the Narratives

All three narratives from each participant were transcribed from the handwritten form to a computerized text format and corrected for grammatical errors for automatic quantitative text analysis. The content of each narrative was then analyzed with the Linguistic Inquiry and Word Count (LIWC) software^3^. The LIWC is an automated software tool which has been frequently used in previous studies and which allows analyzing narratives according to cognitive and emotional factors. The algorithms implemented in the LIWC software counts words according to pre-defined word categories including, e.g., emotional word categories (e.g., positive, negative emotional words), categories of cognitive function words, and pronouns. The output from the LIWC analysis provides units in “words in percent” (frequency of specific words in relation to the total number of words written). In the present study, the latest version of the German dictionary of the LIWC was used for linguistic analysis (Wolf et al., 2008).

Based on previous research and in line with the aims of the study, the following LIWC output variables were assessed and statistically compared across the three narratives separately for each participant sample (healthy participants, depressive participants): (1) category of negative emotional and positive emotional words, (2) category of pronouns including the total amount of referential pronouns (e.g., I, them), first person pronouns singular (e.g., I, my, mine), and first person pronouns plural (e.g., we, us) as separate subcategories, (3) category of cognitive function words comprising cognitive mechanism words (e.g., cause, know, ought), insight (e.g., think, know, consider), and causation words (e.g., because, effect, hence) as separate subcategories, and (4) amount of total words written per narrative.

### Statistical Analysis

The linguistic markers (see section “Analysis and Coding of the Narratives”) were entered into statistical analysis. For the healthy participant sample statistical analysis included OMNIBUS repeated measures analysis of variance (ANOVA) comparing effects as a function of the emotionality (negative, neutral, and positive) of the narratives. Application of the ANOVA designs included testing for sphericity (Mauchly’s test) and correction according to Greenhouse–Geisser where appropriate. In addition, the ANOVA results were further explored by planned comparisons tests (more than two comparisons). Results obtained for the depressive sample of participants (*N* = 7) are reported descriptively only.

## Results

### Content of the Narratives

Given that participants were free to choose the content of positive, negative, and neutral autobiographical events, a descriptive analysis was performed first to determine possible differences in content within each narrative category (positive, negative, neutral) that may affect the analysis of linguistic markers. Descriptive analysis presented in Table 2 showed that healthy participants wrote most often about family events and close relationships that included events of vacation and journeys (79%) when writing expressively about positive events, whereas family events including the death of a beloved one were most frequent when writing about negative events (75%). When writing about neutral autobiographical events, participants most frequently wrote about everyday life activities (82%). Eighteen percent of the healthy participants wrote about personal achievements during positive expressive writing and 14% wrote about personal failures during negative expressive writing. Participants with depression also described most often family events and journeys when writing expressively about positive events (86%) and 14% wrote about personal achievements, whereas reports about death of a beloved one were most frequent when writing about negative events (86%) being followed by writing about personal failures (14%). Also akin to healthy participants, depressed participants chose to write about everyday life activities (85%) when writing about neutral autobiographical events. On average, healthy participants wrote about autobiographical events that were for negative events about 36 months (*SE* = 8.60), for neutral events about 17 months (*SE* = 7.91), and for positive events about 22 months (*SE* = 7.11) in the past. Depressive participants in contrast wrote about negative and positive autobiographical events that on average lay 93 and 82 months in the past (negative: *M* = 92.57 months, *SE* = 22.00; positive: *M* = 82.14 months, *SE* = 21.77) compared to 4 months for neutral events (*M* = 3.71 months, *SE* = 14.34).

**Table 2:** Subjective content of narrated life events in percent (%) illustrated separately for the group of healthy participants and the group of depressive participants.

Event	Healthy participants (*N* = 28)	Depressive participants (*N* = 7)
**Autobiographical life events (%)**		
Negative events in negative narratives	75	86
–Family and close relationships (e.g., death and loss)		
Negative events in negative narratives	14	14
–Personal failures		
Negative events in negative narratives	11	0
–Accidents and disorders		
Positive events in positive narratives	79	86
–Family and close relationships (e.g., journeys, travels, and vacation)		
Positive events in positive narratives	18	14
–Personal achievements		
Positive events in positive narratives	4	0
–Other life events		
Neutral events in neutral narratives	82	85
–Everyday life events (e.g., family, achievements)		
Neutral events in neutral narratives	18	14
–Other life events		

### Word Count Analysis

#### Healthy Participants

##### Use of emotional words across positive, negative, and neutral narratives

**FIGURE 1:**
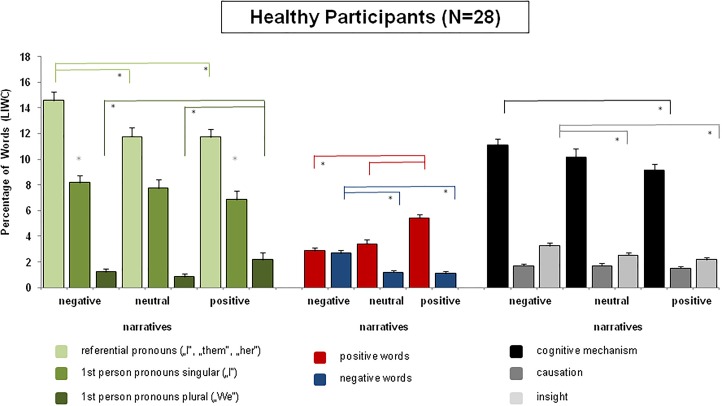
Overview of the linguistic analysis. Figure 1 shows group means and standard errors of the linguistic markers including pronouns, emotional words, and cognitive function words. Values are taken from LIWC. Results are shown for the group of healthy participants across positive, negative, and neutral narratives. Significant results are indicated by stars and explained in detail in the section “*Results*” of the manuscript.

Regarding healthy participants, use of emotional words differed significantly in negative, positive, and neutral narratives. The Omnibus ANOVA revealed significant main effects of the within-subject factors *emotionality* (negative, positive, neutral narratives), *F*(2,54) = 10.02, *p* < 0.0002, and *emotional word use* (positive, negative), *F*(1,27) = 129.98, *p* < 0.0001. The interaction between the two factors was also highly significant, *F*(2,54) = 41.27, *p* < 0.0001, GG-Epsilon = 0.97. Planned comparison tests revealed the following results: regarding negative narratives, healthy participants used more negative words in negative narratives than in positive, *F*(1,27) = 33.86, *p* < 0.0003, or neutral narratives, *F*(1,27) = 32.54, *p* < 0.0001. Moreover, as shown in Figure 1, they used significantly more positive words in negative narratives than vice versa, *F*(1,27) = 98.38, *p* < 0.0001. In negative narratives, use of positive and negative emotional words did not differ, i.e., in negative narratives participants used positive words as often as negative words. Regarding positive narratives, participants used significantly more positive emotional words than negative emotional words, *F*(1,27) = 188.23, *p* < 0.0001. Also, as indicated by the main factor *emotional word use*, this held true across narratives: healthy participants used overall twice as much positive words than negative words. In addition, the amount of positive and negative emotional words differed significantly in neutral narratives: neutral narratives contained significantly more positive emotional words than negative emotional words, *F*(1,27) = 37.84, *p* < 0.0001. Effects are illustrated in Figure 1.

##### Use of referential pronouns – first person singular vs. plural

Use of pronouns was statistically evaluated in an Omnibus ANOVA including the within-subject factors *pronouns* (total amount of referential pronouns, first person singular, first person plural) and *emotionality* (negative, positive, neutral narratives). This revealed significant main effects of the factors *pronouns*, *F*(2,54) = 269.87, *p* < 0.0001, and *emotionality*, *F*(2,54) = 4.14, *p* < 0.02, as well as a significant interaction of the two factors, *F*(4,108) = 5.59, *p* < 0.002, GG-Epsilon = 0.70. Negative narratives of healthy participants contained significantly more referential pronouns (I, them) than neutral, *F*(1,27) = 9.53, *p* < 0.005, or positive narratives, *F*(1,27) = 13.62, *p* < 0.0001. Healthy participants also used more first person pronouns singular during negative compared to positive narratives [negative vs. positive narratives: *F*(1,27) = 4.52, *p* < 0.05]. However, use of first person plural pronouns (we, us) tended to be more pronounced in positive than negative narratives, *F*(1,27) = 4.10, *p* = 0.05. Also, first person plural pronouns were significantly more often used in positive than in neutral narratives, *F*(1,27) = 7.89, *p* < 0.01. Effects are illustrated in Figure 1.

##### Use of cognitive function words

Cognitive function words were split into three subcategories including “cognitive mechanism words,” “causation words,” and “insight words” conform to the categories provided by the LIWC software and effects were statistically compared in an ANOVA including the within-subject factors *emotionality* (positive, negative, and neutral narratives) and *cognitive category* (cognitive mechanisms, causation, and insight). This revealed significant main effects of the factor *emotionality*, *F*(2,54) = 5.62, *p* < 0.006, and of the factor *cognitive category*, *F*(2,54) = 527.70, *p* < 0.001. In addition, a significant interaction between the two factors was observed, *F*(4,108) = 3.00, *p* < 0.05, GG-epsilon = 0.61. *Post hoc* comparisons revealed that healthy participants used significantly more cognitive function words in negative than in positive narratives. Especially, they used significantly more cognitive mechanism words and insight words when writing expressively about negative life events than when writing about positive [cognitive mechanism: *F*(1,27) = 12.14, *p* < 0.002; insight: *F*(1,27) = 27.41, *p* < 0.001] or neutral life events [insight: *F*(1,27) = 331.61, *p* < 0.001)]. Effects are illustrated in Figure 1.

##### Paralinguistic feature analysis (narrative length)

The narratives also differed significantly in length despite the fact that healthy participants were given the same amount of time for writing expressively about positive, negative, or neutral events. Repeated measures ANOVA, *F*(2,54) = 16.69, *p* < 0.001, and *post hoc* comparisons revealed that healthy participants used significantly more words when writing expressively about a negative life event (*M* = 415 words, *SD* = 28) or a positive life event (*M* = 404 words, *SD* = 24) compared to when writing expressively about neutral (*M* = 323, *SD* = 18) life events (each *p* < 0.01). This is in line with the suggestion that narrative length (and number of words written) may reflect the individual significance of a narrated event (e.g., Thompson, 2013). The length of the negative narratives did not differ from positive narratives (*p* = 0.52).

##### Effects of expressive writing on psychosomatic symptoms, bodily sensations, emotional awareness, and mood as indicators of subjective well-being

**FIGURE 2:**
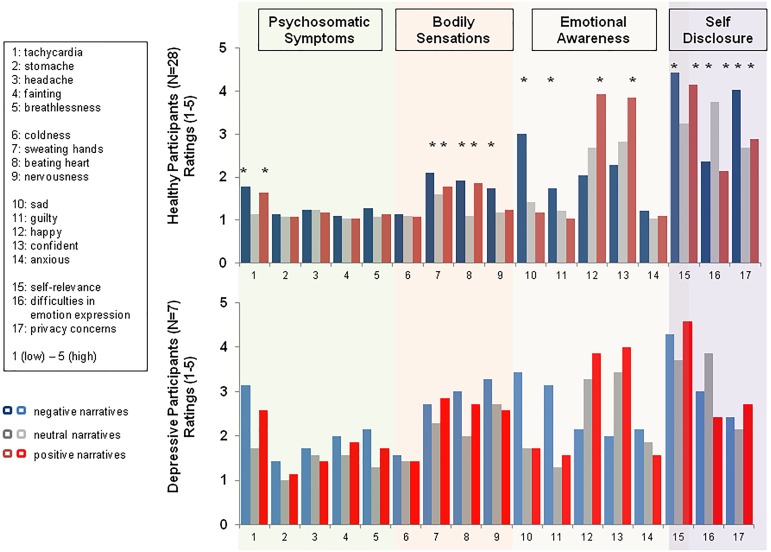
Changes (group means) in self-reported psychosomatic symptoms, bodily sensations, and subjective feelings after positive, negative, and neutral expressive writing. Ratings ranged from 1 (low) to 5 (high) intensity. Data of healthy participants and depressive participants are plotted separately. Significant results are indicated by stars and explained in the section “*Results*” of the manuscript.

*Post hoc* questions regarding psychosomatic symptoms perceived changes in bodily sensations and emotional awareness were statistically tested, each within a one-way repeated measure ANOVA containing the within-subject factors *psychosomatic symptoms* or *bodily sensations* or *emotional awareness* and *emotionality* (positive, negative, or neutral narratives). Overall, healthy participants reported only little changes in psychosomatic symptoms after expressive writing. Nevertheless, there were significant differences in self-reported psychosomatic symptoms between narratives [*emotionality*: *F*(2,54) = 8.00, *p* < 0.001; *psychosomatic symptoms* × *emotionality: F*(8,216) = 29.01, *p* < 0.0001, GG-Epsilon = 0.51]. As shown in Figure 2, healthy participants reported stronger changes in symptoms related to tachycardia after writing about negative and positive events compared to neutral events (each *p* < 0.002). Likewise, writing about negative and positive life events compared to writing about neutral events was associated with a significant increase in self-reported bodily sensations [*emotionality: F*(2,54) = 14.35, *p* < 0.0001, negative vs. neutral, *F*(1,27) = 14.36, *p* < 0.0001, and positive vs. neutral, *F*(2,54) = 19.02, *p* < 0.0001]. Participants rated bodily sensations like sweating hands and a beating heart as more intense after positive and negative writing compared to neutral writing, whereas they felt more nervous after negative than positive or neutral writing (*bodily sensation* × *emotionality: F*(8,216) = 29.01, *p* < 0.0001, GG-Epsilon = 0.51). In addition, expressive writing was associated with a significant increase in emotional awareness: effects differed across narratives, [*emotional awareness* × *emotionality: F*(8,216) = 29.01, *p* < 0.0001, GG-Epsilon = 0.51]. As shown in Figure 2, after writing expressively about negative life events healthy participants reported feelings of sadness, guilt, and fear whereas feelings of happiness and confidence were most pronounced after writing expressively about positive life events, both in comparison to the negative and neutral writing conditions (all *p* < .05). Regarding changes in mood (positive and negative affect as assessed by the PANAS state questionnaire), repeated measures ANOVA containing the main factors *condition* (baseline, negative, positive, and neutral narrative) and *affect* (positive affect vs. negative affect) showed that affect changed across the writing conditions [*condition*: *F*(3,81) = 7.96, *p* < 0.0001]. However, compared to the baseline measurement assessed prior to the experiment, expressive writing did not lead to significant increases or decreases in positive affect or in negative affect, (respectively, *condition* × *affectivity:* n.s., *p* = 0.17). Effects are summarized in Figure 2.

##### Manipulation check: self-disclosure and privacy concerns

Healthy participants reported no difficulties in emotion expression. On the 5-point-Likert scales they rated emotion expression in the negative (*M* = 2.36, *SE* = 0.23) and positive (*M* = 2.14, *SE* = 0.21) expressive writing conditions as less difficult compared to neutral writing (*M* = 3.76, *SE* = 0.27). In addition, healthy participants reported that privacy concerns and concerns about feeling safe matter for self-disclosure, especially for self-disclosure during negative expressive writing (negative: *M* = 4.04, *SE* = 0.23; positive: *M* = 2.90, *SE* = 0.24; neutral: *M* = 2.67, *SE* = 0.29). Writing about negative and positive autobiographical events was experienced as highly self-relevant and personal (negative: *M* = 4.42, *SE* = 0.17; positive: *M* = 4.14, *SE* = 0.17; neutral: *M* = 3.25, *SE* = 0.24).

##### Exploratory descriptive analysis: depressive participants (N = 7)

**FIGURE 3:**
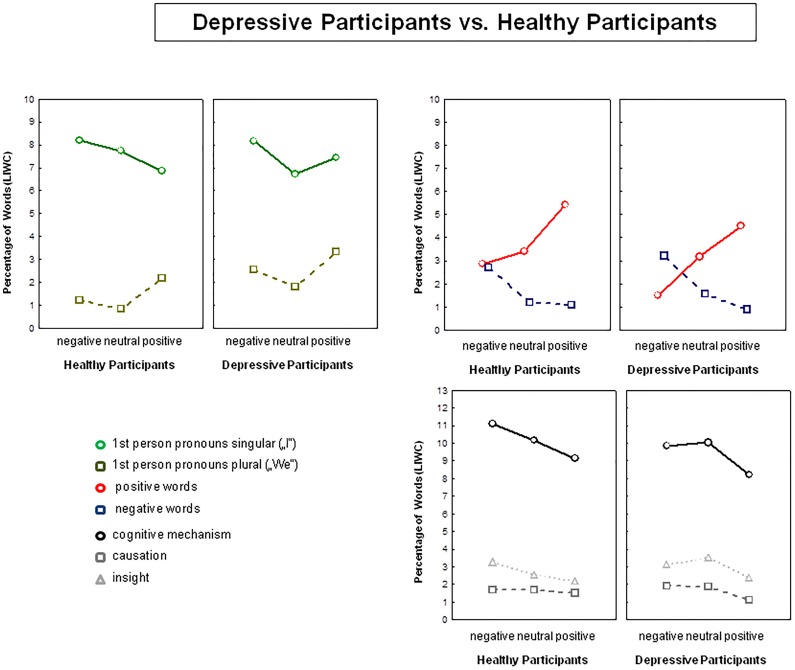
Linguistic data (group means) showing results from the descriptive analysis of the data comparing depressive participants and healthy participants in linguistic markers including pronouns, emotional words, and cognitive function words. Values are taken from LIWC. Results are shown for the positive, negative, and neutral narratives.

As illustrated in Figure 3, for depressive participants, descriptive comparisons did not support the hypotheses of (a) a more rigorous use of first person pronouns singular specifically in negative narratives, or (b) a more rigorous use of negative words in negative narratives compared to healthy controls. Akin to healthy controls, depressive participants seem to use more negative words in negative narratives than in positive or neutral narratives and they also used more positive words in positive and neutral narratives. However, compared to healthy participants, negative narratives of depressive participants contained more negative emotional words than positive emotional words (see Figure 3) and depressive participants wrote significantly longer negative than positive or neutral narratives suggesting a higher significance of negative than positive events in depressive participants and therefore supporting a negativity bias in negative expressive writing. In summary, the most obvious differences in the narratives written by depressive participants vs. healthy participants were (a) a lack of use of positive words in negative narratives and (b) no increased use of cognitive mechanism or insight words specifically in negative narratives. Regarding post-writing symptoms, as shown in Figure 2, the rating patterns of depressive participants did not differ from those of healthy participants. Akin to healthy participants, depressive participants reported an increase in happiness and self-confidence after writing about positive life events and an increase in feelings of sadness, guilt, and anxiety after writing about a personally challenging negative event. Depressive participants also reported no difficulties in emotion expression. In the manipulation check, emotion expression in the negative and positive expressive writing conditions were rated as less difficult compared to neutral writing (negative: *M* = 3.00, *SE* = 0.45; positive: *M* = 2.42, *SE* = 0.92; neutral: *M* = 3.86, *SE* = 0.53). Privacy concerns and concerns about self-disclosure were rated equally important across the three writing conditions (negative: *M* = 2.42, *SE* = 0.49; positive: *M* = 2.71, *SE* = 0.45; neutral: *M* = 2.14, *SE* = 0.53). Akin to healthy participants, depressive participants experienced writing about positive and negative autobiographical events as most self- and personally relevant (negative: *M* = 4.29, *SE* = 0.34; positive: *M* = 4.57, *SE* = 0.32; neutral: *M* = 3.71, *SE* = 0.47).

## Discussion

The present study investigated changes in emotion expression and in subjective well-being in healthy participants during expressive writing about positive, negative, and neutral autobiographical life events. Changes in emotion expression were assessed by analyzing linguistic markers that according to previous expressive writing research provide a window into the writer’s self, his/her most private feelings, and autobiographical memories. Linguistic analysis comprised linguistic categories that relate to the emotionality and the self-reference of the writing and to the way the writer perspectively accesses, mentally structures, and represents and possibly reappraises his/her emotional experiences. Therefore, special focus was given to the analysis of emotional words, referential pronouns, and cognitive function words as linguistic markers of positivity/negativity, self-reference, and cognitive reappraisal.

Regarding the interplay between words and emotionality, negative narratives of healthy participants contained more negative emotional words than did positive or neutral narratives. Moreover, positive narratives contained more positive emotional words than negative narratives. All in all the proportional use of emotional words as a function of the emotionality of the narratives accords with the findings from previous studies on expressive writing investigating mostly English speaking populations. The present study validates these findings for native speakers of German and for analysis based on the German dictionary provided by LIWC (Wolf et al., 2008).

### Negativity Bias vs. Positivity Bias

Further analysis revealed significant differences in the use of positive emotional words as well as in the use of referential pronouns including first person personal pronouns (singular vs. plural) and in the use of cognitive function words: negative narratives contained not only on average more negative words than positive or neutral narratives; they also contained significantly more positive emotional words compared to negative emotional words in positive narratives. Furthermore, healthy participants also used significantly more cognitive function words, cognitive mechanism and insight words in particular, in negative than in positive or neutral narratives. They also used more referential pronouns in negative than in positive or neutral narratives. Regarding self-reference, participants used more first person personal pronouns singular in negative narratives compared to positive narratives.

On the one hand, these observations seem to support a negativity bias and a larger self-focus in healthy participants during negative than during positive autobiographical writing which would be in line with the idea that use of certain linguistic markers is content-specific and context-dependent. Furthermore, the increase in positive words and cognitive mechanism and insight words in negative narratives also lends support that particularly during negative writing healthy participants aimed to reappraise their experiences in a positive and insightful way (Tausczik and Pennebaker, 2011). The use of cognitive mechanism words and insight words during negative expressive writing indicates that healthy participants provided complex information and reasoning about negative events. Research in positive psychology suggests that healthy people try to give their negative life events a redemptive character by finding the positive aspects of the negative life events (e.g., McAdams et al., 2001). Theoretically, it has also been assumed that healthy people prefer searching for positive information and avoid thinking of negative information to facilitate individual well-being (Baumeister et al., 2001). Hence, using positive words and cognitive function words during negative expressive writing could reflect positive health-oriented mental strategies of healthy participants.

On the other hand and in line with the hypothesis of a positivity bias, writing about positive events was also characterized by a high rate of positive words. Also writing about neutral events was associated with a positivity bias (more positive than negative words). As shown in Figure 1, participants used on average four times as many positive than negative words, supporting a universal positivity bias (e.g., Dodds et al., 2015; Diener et al., 2018). According to some authors, this overall positivity bias reflects mental health and well-being (Schwartz, 1997).

### Negativity Bias vs. Positivity Bias and Self-Reference

Besides a negativity and a positivity bias, specifically positive narratives contained more pronouns of the first person plural (“we”) compared to negative narratives. As already mentioned above, negative narratives contained a higher rate of first person pronouns singular compared to positive narratives but no comparable increase in the use of first person pronouns plural. Hence, healthy participants seem to spontaneously associate negative autobiographical events more strongly with the individual self (“I”, “Me”) and may extend positive events from the individual to the social self (“We”). Several studies and theoretical research (Brewer and Gardner, 1996) could already demonstrate that individuals broaden their self-focus to significant and close others under certain conditions such as when experiencing positive events or wins that can be shared with others (e.g., Chung and Pennebaker, 2008) or when passionately in love (e.g., Aron and Aron, 1986; Meixner and Herbert, 2018). In addition, some studies suggest that this broadening of one’s self-focus can be associated with an increase in the use of first person personal pronouns [Pennebaker and Chung (2007) for an overview]. The present findings accord with these observations and confirm them during expressive writing about positive autobiographical events. That healthy participants show changes in self-focus from an egocentric to a we-centered relational perspective during one single session of expressive writing, positive writing in particular, extends previous research in important ways. Whether writing repeatedly about positive life events can shift self-other boundaries continuously from an egocentric to a relational perspective and whether this will be expressed during writing by changes in self-awareness from first person singular to first person plural (“We”) should therefore be examined and investigated in future studies.

### Expressive Writing and Depressive Symptoms

Preferential use of positive words in negative narratives was absent in participants reporting former depressive disorder and mild depressive symptoms. As shown in Figure 3, negative narratives of depressive participants contained more negative than positive words and depressed participants wrote significantly longer negative than positive or neutral narratives supporting an overall depression-related negativity bias. Interestingly, there was no indication of a more prominent use of cognitive mechanism or insight words in negative narratives suggesting in depressive participants little evidence for cognitive reappraisal of negative life events. These differences between depressed and healthy participants – albeit descriptive – cannot be accounted for by differences in writing content. Analysis of negative, positive, and neutral story contents revealed that depressive and healthy participants wrote about the same negative, positive, and neutral topics (death of a beloved one, holiday travels and vacation, and ordinary life events). The descriptive comparison between healthy participants and depressive participants is preliminary and the results in depressive participants are limited due to the very small sample size (*N* = 7 depressive participants). Yet, together, the results observed in healthy participants and in depressive participants support the assumption that linguistic markers of self-focus (pronouns), emotionality (emotional words), and cognitive restructuring (function words) are affective and cognitive state and trait markers of emotion expression; sensitive to context (i.e., the emotional content of the writing) and to intra- and interindividual differences in emotion expression.

### Short-Term Effects of Expressive Writing on Psychosomatic Symptoms, Mood, and Awareness of Bodily Symptoms

Regarding short-term effects of expressive writing, negative and positive writing changed subjective well-being immediately after the writing condition. Consistent with previous research, in the present study subjective well-being decreased immediately after having written expressively about an emotionally distressing autobiographical event. This was associated with feelings of sadness and self-reported psychosomatic symptoms, tachycardia in particular, and with bodily sensations of sweating, a beating heart and nervousness, i.e., emotion-unspecific physiological sensations related to sympathetic arousal. Writing about positive autobiographical events was accompanied by happiness and self-confidence. In addition, compared to neutral writing, writing about positive autobiographical events also increased bodily sensations (beating hearts) in healthy participants suggesting better emotional awareness for bodily signals after expressive writing regardless of the emotional content. However, compared to the pre-writing baseline condition neither did negative nor positive writing change self-reported positive or negative affect. This implies that the aforementioned changes in subjective well-being are temporally restricted to instantaneous changes in emotional and bodily awareness. Nevertheless, these changes fluctuate with how people put their positive and negative memories into words.

### Limitations and Future Directions

The present study shed further light onto the question of how language and emotion interact during expressive writing. The linguistic markers under investigation confirmed and extended a number of observations from previous research. But, the present study also has restrictions that need to be taken into consideration in future research.

First, the results of the present study hold for a single session of expressive writing. Whether the present results can be generalized to settings other than expressive writing (e.g., natural language use) needs to be determined in future studies because entirely different patterns may be obtained for the same person and class of words during analysis of natural language processing (e.g., Rodriguez et al., 2010). Second, the linguistic analysis confirm an overall positivity bias in healthy subjects, an extension from self- to we-reference especially during positive writing and reappraisal of negative events – as was indicated by an increased use of positive words and of cognitive function words in negative narratives. The latter two patterns could on a descriptive level both not be observed in depressive subjects. As already mentioned, the results in the depressive sample rely on a total of seven participants, all reporting mild depressive symptoms and six reporting former diagnosis of major depressive disorder. The results may therefore not be representative for all depressive patients. Also, they cannot give any recommendation of whether a single session of negative or positive expressive writing can be an effective tool for depressive counseling or depression-related therapeutic intervention. Regarding therapeutic effects, a recent meta-analysis summarizing the results of more than 150 studies could not find evidence in support of an effectiveness of brief, self-directed expressive writing as an intervention against, for instance, depressive symptoms in physically healthy adults or depressed participants (e.g., Reinhold et al., 2018).

Nevertheless, regarding immediate changes in subjective well-being, the present results show that putting feelings into words is well capable of evoking bodily sensations and changes in emotional awareness at least temporarily. Regarding the expression of feelings during writing none of the participants reported difficulties in getting in touch with their feelings during positive and negative writing. Thus, expressive writing about positive and negative life events is not a disembodied cognitive process but an embodied process facilitating the interplay between mind and body. Regarding the willingness to get in touch with one’s own self during writing, all participants reported that feeling safe and private during writing had a significant impact on the extent of self-disclosure particularly when reporting about the most private negative autobiographical events. These reports should be taken seriously as participants’ privacy concerns might matter especially when assessing data online via the Internet, e.g., during web-based interventions and hence in situations where individual anonymity is often taken for granted. In addition, effects may vary from each single memory to memory and with age and gender. Therefore, the present data in healthy participants may only speak for young and healthy adults.

There was an overrepresentation of women in both samples of participants. Thus, regarding gender-effects the present results should be interpreted with caution. As far as the topics chosen for expressive writing are concerned, the present study achieved high between-subject consistency. Participants selected above chance the same topics for expressive writing for why the topics chosen in the present study may serve as prototypical exemplars of negative, positive, and neutral life experiences. Nevertheless, especially with regard to negative writing one cannot exclude that writing expressively about less emotionally distressing events and topics will yield different results. So far, the results are consistent with previous studies asking participants to write about the most emotionally distressing experience.

Taking these restrictions into consideration the present results suggest that putting feelings into words may be as imperative for emotion expression, health, and well-being as non-verbal emotion expression. Analyzing people’s writings and the words they use may therefore constitute a valid means for the investigation of the cognitive and affective mental representations of one’s own and another person’s self. Regarding future investigations, quantitative approaches as the one applied in the present study and promoted in previous expressive writing studies may also benefit from including qualitative and hermeneutic methods and theoretical approaches highlighting that language can be both, (a) a tool providing insight into our self, our actions, thought, and feelings and (b) a means of storytelling and metacognition, i.e., allowing subjects to move on from naive to deeper understanding of their own narrative.

## Data Availability Statement

The raw datasets for this manuscript are not publicly available because informed consent of the participants restricts data sharing via public repositories. However, the datasets for this manuscript (group means) can be made available by the authors, without undue reservation, to any qualified researcher upon email request.

## Author Contributions

CH developed, conceptualized, designed, and supervised the study. EB collected, recorded, and analyzed the data. CH and EB wrote the first drafts of the manuscript. CH wrote the manuscript, re-analyzed, and approved the data. CH and EB created tables and CH created the figures. RR helped in data collection of the depressive participants and supervised the study with regards to safety procedures for depression. CH, EB, and RR revised and approved the manuscript for submission.

## Conflict of Interest Statement

The authors declare that the research was conducted in the absence of any commercial or financial relationships that could be construed as a potential conflict of interest.

## Footnotes

1 The German translation and adaption of the PSS (Pennebaker, 1982), the German translation and adaption of the writing instructions, and the questions created in this study and used in the manipulation check are available from the corresponding author upon request.

2 https://www.uni-ulm.de/einrichtungen/ethikkommission-der-universitaet-ulm/

3 http://liwc.wpengine.com/
